# XRD and Molecular Dynamics Insights into Lattice Behavior of Oxide Nanocatalysts: The Case of CeO_2_

**DOI:** 10.3390/nano16050333

**Published:** 2026-03-06

**Authors:** Sirisha Subbareddy, Marcelo Augusto Malagutti, Himanshu Nautiyal, Narges Ataollahi, Paolo Scardi

**Affiliations:** 1 Department of Civil, Environmental, and Mechanical Engineering, University of Trento, 38123 Trento, Italy; sirisha.subbareddy@unitn.it (S.S.); narges.ataollahi@unitn.it (N.A.); 2 Department of Physics, School of Physical and Biological Sciences, Manipal University Jaipur, Jaipur 303007, Rajasthan, India; himanshu.nautiyal@jaipur.manipal.edu

**Keywords:** X-ray powder diffraction, thermal diffuse scattering, molecular dynamics simulations, metal oxide catalyst, surface chemistry

## Abstract

Nanocrystalline CeO_2_ exhibits size-dependent lattice distortions linked to defect chemistry and surface effects. However, the relationships between the oxidation state, surface interactions, and nanoparticle structure remain unclear in the existing literature, particularly when inferred from conventional nanoparticle diffraction techniques, including powder X-ray diffraction. As a result, the atomistic origin of lattice expansion or contraction with the crystallite size of ceria nanoparticles is still debated. Here, synchrotron X-ray powder diffraction data are analyzed using Rietveld refinement supported by advanced peak profile modeling based on whole powder pattern modeling (WPPM), including thermal diffuse scattering (TDS). The latter provides direct access to information on lattice dynamics. Indeed, we simultaneously determine the size distributions of crystalline domains and their atomic displacements, which are then compared and quantitatively validated with molecular dynamics (MD) simulations. Reactive MD simulations further reveal that vacancy-rich surfaces induce lattice contraction at small particle sizes under vacuum, whereas water adsorption causes surface hydroxylation and lattice expansion. These results explain lattice parameter variations in nanocrystalline ceria through the interplay of surface chemistry and environment. This insight is critical for the correct interpretation of diffraction-derived structural parameters in oxide nanocatalysts used in redox and oxygen storage applications.

## 1. Introduction

Diffraction methods are the primary tools for determining crystal structures, as sharp Bragg reflections describe the long-range periodic order of atoms. In addition to these discrete peaks, diffraction patterns contain scattering from dynamic atomic displacements, which reduce Bragg peak intensities and shift part of the scattered intensity into TDS. This separation is convenient, as Bragg peaks describe the average crystal structure, while TDS provides additional information about lattice vibrations and interatomic forces, representing a second-order contribution to the diffraction signal [1]. Two principal methodologies exist to analyze these contributions in XRPD. In total scattering analysis, Bragg and diffuse intensities are treated on an equal footing, either through Fourier transformation into the pair distribution function (PDF) [2] in real space or via reciprocal-space modeling with the Debye scattering equation (DSE) [3,4]. Alternatively, structure refinement approaches model Bragg and diffuse components separately. In this context, there are two main models to describe the TDS: (i) the Warren model, which captures long-range vibrational effects within the limits of Debye’s theory of specific heat [5], and (ii) the Sakuma formalism, treating atomic pairs as correlated oscillators and which is therefore suitable for highlighting short-range dynamical correlations (local dynamics) [6]. The latter is preferred here, as it is not constrained by the assumptions underlying the Debye specific heat model, which provides only an approximate description of local lattice dynamics.

This framework is particularly relevant to many of the metal oxide nanocatalysts. CeO_2_ is a good example, which is widely studied for its redox behavior [7] and high oxygen storage capacity [8]. It plays a central role in energy storage and conversion applications, including solid oxide fuel cells [9], thermochemical water splitting [10], CO oxidation [11], photocatalytic hydrogen production [12], and electrochemical CO_2_ reduction [13], where its ability to shuttle oxygen and stabilize reactive intermediates is critical to performance. In these applications, catalytic performance is strongly influenced by surface area, as a high surface-to-volume ratio increases the number of accessible active sites. This requirement is typically achieved by synthesizing CeO_2_ nanocrystals with dimeters on the order of 10 nm or smaller, where the abundance of surface oxygen vacancies [14] and Ce^3+^ species enhances catalytic activity [15]. However, structural characterization of CeO_2_ nanoparticles is challenging due to the coexistence of Ce^3+^/Ce^4+^ states, surface vacancies, and the presence of adsorbates. These factors strongly influence diffraction-derived structural parameters and complicate the separation of intrinsic size effects from defect- and surface-driven contributions. Consequently, conventional crystallite size modeling via XRPD does not adequately capture the crystallite size distribution, both when using the Williamson–Hall method and Double-Voigt peak profile fitting, as they rely on arbitrary assumptions that are unsuitable for the case of nanocrystalline powders. In particular, Williamson–Hall attempts to extract size and strain from peak width alone [16], while the Double-Voigt method imposes a predefined line profile shape that is not well suited to capture realistic size–strain effects in nanocrystalline powders [17,18]. Furthermore, several structural and dynamic parameters vary with crystallite size, including the lattice parameter [19], microstrain, and Debye–Waller coefficients [20]—which are proportional to the atomic mean square displacement (MSD). Such effects can be addressed within the WPPM [21], which allows for the refinement of a crystallite shape and size distribution through histogram-based approaches combined with appropriate microstrain modeling. In addition, the dynamics of the system can be analyzed by modeling the diffuse component using the Sakuma TDS model [20] or via PDF.

Complementary to diffraction-based methods, molecular dynamics (MD) simulations and DFT calculations provide atomistic and electronic perspectives on CeO_2_. MD simulations reveal how lattice vibrations, oxygen diffusion, and anharmonic displacements evolve with temperature, while DFT captures the charge redistribution across the Ce–O network [22]. While these computational approaches generate valuable structural and electronic information, they are often applied independently and interpreted separately from diffraction results. Diffraction analyses typically stop at quantifying disorder, MD studies emphasize mobility [23], and DFT studies highlight electronic redistribution [24,25], but rarely are these threads woven together to explain how lattice vibrations, electronic localization, and defects jointly influence redox behavior [26]. Given that the redox functionality of CeO_2_ hinges precisely on oxygen dynamics and vacancy formation, there is a clear need for an integrated framework that unites TDS-informed diffraction, MD simulations, and electron localization function (ELF) descriptors from DFT.

In this work, we present an integrated analysis that combines WPPM and TDS to simultaneously resolve crystallite size distributions and lattice dynamics from synchrotron XRPD data. Displacement correlation coefficients obtained using the Sakuma model are assessed against MD simulations, establishing a link between reciprocal-space diffraction observables and real-space vibrational behavior. Reactive MD simulations based on the ReaxFF formalism and DFT calculations were employed to explicitly examine vacancy-rich and hydrated CeO_2_ nanoparticles, providing size-dependent structural descriptors that capture the influence of surface chemistry and redox behavior. This combined approach integrates crystallite size distributions and lattice dynamics within a single diffraction framework while enabling mechanistic interpretation of chemically driven structural responses, offering a methodology that is applicable to metal oxide nanocatalysts.

## 2. Experimental and Computation Details

### 2.1. Experimental Details

CeO_2_ nanoparticles were produced via sol–gel method following a procedure described elsewhere [27]. Three samples are considered in the present work, produced with varying calcination temperatures in air: 300 °C, 500 °C, and 600 °C.

Synchrotron measurements were performed in the ESRF ID31 beamline. A 0.06325(5) nm wavelength was employed, estimated using a SRM 640c silicon standard [28]. LaB_6_ standards (660a) [29] were used for determining the instrumental resolution function (IRF) using TCHZ-like profiles. A 0.3 mm capillary was employed, spun during the measurement to increase the statistics. Two measurements from 2° to 90° were performed, with a 4° min^−1^ rate. Empty capillary was measured in the same conditions. The Rietveld + WPPM + TDS and PDF analyses were performed using TOPAS software V8 and macros [20,30,31].

### 2.2. Atomistic Simulations

#### 2.2.1. Buckingham and Coulomb Potential Simulations

The molecular dynamics simulations of CeO_2_ were performed using the LAMMPS package [32]. The system was constructed from an input structural file (PDF # 00-004-0593) and replicated to generate a supercell of 31 × 31 × 31, using periodic boundary conditions (PBCs). A Buckingham potential was used with values given in Ref. [33] with a calculation cutoff distance of 10 Å. The integration of equations of motion was carried out using the velocity–Verlet algorithm with a timestep of 2 fs. The system was first minimized with the Hessian-free truncated Newton method to relax any residual forces, after which it was equilibrated. Initial velocities were assigned using a Boltzmann distribution for the following temperature simulations: 300, 600, 1400, 1600, 1800, 2000, and 2200 K. Equilibration involved a two-step process: initial thermalization under isothermal–isobaric (NPT) conditions at 300 K and 1 atm for 40 ps to relax the simulation cell, followed by an additional 40 ps of isothermal (NVT) dynamics at 300 K to stabilize temperature fluctuations. Temperature and pressure coupling were achieved through Nosé–Hoover thermostat and barostat with damping constants of 2 ps. Finally, the production run was carried out in the microcanonical (NVE) ensemble for 40 ps.

The last 100 frames of the NVE were selected to analyze the thermal behavior. Perfectly spherical nanospheres of 16 nm in diameter were carved out from the simulation box and used to calculate the DSE using Debyer [34], producing the simulated X-ray patterns. A C++ code was developed to estimate the correlation coefficients directly from these 100 frames by estimating the MSD and MSRD relative to the first frame of the simulations.

#### 2.2.2. ReaxFF Simulations of Water–Ceria Surface Interactions

This study investigates the influence of crystallite size on the average lattice parameter of pure and hydroxylated CeO_2_ under vacuum conditions. Two sets of perfectly spherical CeO_2_ nanospheres with diameters of 2, 3, 4, 5, 6, 8, 10, 15, and 20 nm were first carved from the simulation box. In the first set, CeO_2_ cores were coated with a thin Ce_2_O_3_ shell (0.5 nm thick), where 10% of the surface oxygen atoms were removed to introduce vacancies. This vacancy concentration was chosen to represent a moderately reduced surface, consistent with experimental and computational studies of nanoceria under vacuum or non-oxidizing conditions, while allowing vacancy-driven surface relaxation effects to be isolated without inducing bulk-like reduction [35]. These individual core–shell nanoclusters were simulated using ReaxFF force fields (interactive potentials).

In the second set, the core–shell nanoclusters were further functionalized with a monolayer of water, 2.7 Å thick, with the inner shell radius defined as $R_{in}=R_{NP}+3.0$ Å, where $R_{NP}$ represents the maximum radial extent of the CeO_2_ nanoparticle measured from its geometric center. This offset ensures that water molecules are initially positioned outside the short-range repulsive region of the interatomic potential, consistent with typical physisorption separations on the order of ~3 Å. The initial configuration therefore corresponds to molecularly adsorbed water; hydroxylation and dissociation occur only during subsequent ReaxFF equilibration. Water molecules were added as an explicit spherical shell around the nanoparticle using a geometry-based builder (rigid H_2_O, random orientations) with overlap rejection [36]. The number of water molecules in each “monolayer” was not arbitrarily chosen but estimated from the shell volume assuming bulk water density (1.0 g cm^−3^), i.e., $N=\rho VN_{A}/M$ (N is the number of water molecules, ρ is the density of water, V is the volume of the spherical shell, $N_{A}$ is the Avogadro’s number and M is the molar mass of water). All reactive molecular dynamics simulations were performed using the LAMMPS package (7 Feb 2024—Update 1) with KOKKOS GPU (version 4.6.2) acceleration, employing ReaxFF parameters specifically optimized for Ce–O–H systems [25].

The simulation cells, comprising CeO_2_ nanoparticles with surface oxygen vacancies and explicit water molecules, were modeled under PBC conditions. Charge equilibration was performed every timestep using the fix qeq/ReaxFF scheme. Initial atomic coordinates were energy-minimized using the conjugate gradient algorithm to remove unfavorable contacts. Subsequently, systems were equilibrated in the NVT ensemble at 300 K using a Nosé–Hoover thermostat with a damping constant of 100 fs and a timestep of 0.1 fs for 100,000 steps (≈10 ps). Production runs were then conducted in the NVE ensemble for the same duration to ensure energy conservation and to collect trajectory data. Atomic positions, velocities, and partial charges were recorded every 500–1000 timesteps for detailed structural and dynamical analyses. The last 100 frames of the NVE trajectories were selected to analyze the diffraction patterns using the DSE.

#### 2.2.3. DFT Studies

DFT calculations were carried out with Vienna ab initio Simulation Package (VASP 6.2.1), using the using the PBE generalized gradient exchange–correlation functional and PAW pseudopotentials. Exchange–correlation effects were treated within the PBE generalized gradient approximation. The calculations were performed on bulk CeO_2_, modeled as a 2 × 2 × 2 fluorite supercell containing 32 Ce and 64 O atoms. A plane wave energy cutoff of 520 eV was found sufficient for convergence, and Brillouin zone sampling was carried out on a Γ-centered 2 × 2 × 2 Monkhorst–Pack grid. Structural relaxation was performed using a conjugate gradient algorithm, allowing both atomic positions and lattice parameters to vary, until the electronic self-consistency criterion of 1 × 10^−7^ eV was reached. Electronic occupancies were treated using Gaussian smearing with a width of 0.01 eV, and an additional support grid was employed to improve force accuracy.

The electron localization function (ELF) was evaluated from the converged charge density to examine the electronic connectivity of the oxygen sublattice and the Ce–O bonding environment. ELF isosurfaces were generated and visualized using the VESTA software (4.6.0) package [37]. In the context of this work, DFT calculations are used to provide an electronic structure reference that complements the diffraction and molecular dynamics analyses, supporting the interpretation of anisotropic atomic displacement correlations and surface chemistry effects discussed in the main text.

## 3. Theoretical Basis

### 3.1. Thermal Diffuse Scattering Models

The intensity scattered from a crystalline powder can be expressed as the sum of two terms:

(1) $$I=I_{B}+I_{D}$$

where $I_{B}$ represents the Bragg component associated with the crystalline structure of the system and $I_{D}$ accounts for the diffuse scattering originating from atomic disorder. Historically, the Einstein model, which assumes uncorrelated atomic vibrations, provided the bases for the development of the Debye model for TDS [38]. Within this approximation, the diffuse scattering intensity can be expressed as

(2) $$I_{D}\left(Q\right)=k(Q)\sum_{s}n_{s}\;{\left|f_{s}\left(Q\right)\right|}^{2}\left(1-e^{-2M_{s}\left(Q\right)}\right)$$

where $Q=4\;\pi \sin\left(\theta \right)/\lambda$ is the modulus of the momentum transfer due to the X-ray scattering, k(Q) groups constants and known functions of *Q* to correct for aberrations and geometric effects (e.g., Lorentz-Polarization factor), $f_{s}$ is the atomic form factor of species s, and $n_{s}$ is the number of atoms s per unit cell. $M_{s}\left(Q\right)=B_{s}\;{\left(Q/4\pi \right)}^{2}$ is the exponent of the Debye–Waller factor, and $B_{s}$ the Debye–Waller coefficient of atoms *s*. Naturally, as temperature increases, atomic displacements grow, which in turn increase the TDS component and reduces the Bragg scattering.

Although this captures the redistribution of intensity with thermal disorder, it neglects the fact that vibrations are not independent, as near-neighbor atoms are coupled through interatomic forces. To account for these correlations, models such as the Sakuma formalism [39] introduce explicit displacement correlation functions into the diffuse term. Within this framework, the intensity becomes

(3) $$I_{D}\left(Q\right)=k\left(Q\right)\left[\sum_{s}n_{s}f_{s}f_{s}^{*}\left(1-o_{i}\;e^{-2M_{s}}\right)+\sum_{s}\sum_{s^{\prime }}n_{s}f_{s}f_{s^{\prime }}^{*}\left(o_{i}\;e^{-\left(M_{s}+M_{s^{\prime }}\right)\left(1-\lambda_{r_{{ss}^{\prime }}}\right)}-e^{-\left(M_{s}+M_{s^{\prime }}\right)}\right)Z_{ss^{\prime }}\frac{\sin\left(Qr_{ss^{\prime }}\right)}{Qr_{ss^{\prime }}}\right]$$

where $r_{ss^{\prime }}$ is the separation between atoms s and $s^{\prime }$, $Z_{ss^{\prime }}$ is the number of sites belonging to the *s*’th neighbor around an *s*th site, $o_{i}$ is the occupancy factor, and $\lambda_{r_{ss^{\prime }}}$ is the correlation coefficient. The correlation coefficients are given by

(4) $$\lambda_{r_{{ss}^{\prime }}}=\frac{\left\langle \Delta r_{s}\cdot \Delta r_{s^{\prime }}\right\rangle }{\left\langle {\left|\Delta r_{s}\right|}^{2}\right\rangle +\left\langle {\left|\Delta r_{s^{\prime }}\right|}^{2}\right\rangle }=\frac{1}{3}\frac{{\mathrm{MSRD}}_{ss^{\prime }}}{{\mathrm{MSD}}_{s}+{\mathrm{MSD}}_{s^{\prime }}}$$

where $r_{s}$ is the atom s position vector, $\Delta r_{s}$ is the deviation from the reference system (the reference system can be the average value or the position of the atom in the first frame), and MSRD denotes the mean squared relative displacement of the pair. Values of $\lambda_{r_{ss^{\prime }}}$ range from +1 to −1, describing rigid bond-like correlations and out-of-phase motions, respectively. Positive values correspond to acoustic modes of vibrations whereas negative values are associated with optical vibrational modes. If $\lambda_{r_{{ss}^{\prime }}}=0,$ no correlation is present and Equation (3) falls into the case of Equation (1). Full derivation of these equations is given in Refs. [20,39,40]. These coefficients can be estimated using the C++ routine described in Supplementary Note S1. In addition, guidelines for the use of the TDS macros in TOPAS based on previous works (vide Refs. [20,39,40]) are provided in Supplementary Note S2.

### 3.2. Crystallite Size Histogram Distribution

The lattice parameter of ceria is strongly influenced by both the oxidation state and the crystallite size [41,42,43]. To account for this dependence, a size-dependent lattice parameter is introduced by describing the crystallite population through a histogram-based size distribution, in which a specific lattice parameter is assigned to each size bin D. In WPPM, the intensity $I_{S}(Q)$ for a particular scattering vector modulus is given by its Fourier transform due to size effects, $A^{S}(L)$, given by [21,31]:

(5) $$I_{S}\left(Q\right)=k\left(Q\right)\int A^{S}(L)\exp\left(2\pi i\;L\;d_{hkl}^{*}\right)\;dL$$

where Q is the reciprocal space length in Bragg condition and L is the Fourier length. Similarly to Equations (1) and (3), k(Q) includes Q-related intensity corrections (e.g., Lorentz-Polarization, squared modulus of the structure factor, etc.). The $A^{S}\left(L\right)$ term is given by the following relationship for a perfect sphere of diameter *D* [44]:

(6) $$A_{sph}^{S}=1-\frac{3}{2}\frac{L}{D}+\frac{1}{2}\frac{L^{3}}{D^{3}}\;(0\leq L\leq D)$$

With the following macro in TOPAS it is possible to model the domain size contribution in a powder of identical spheres:

macro WPPM_Sphere(RRc, RRv)
{
    #m_argu RRc
   If_Prm_Eqn_Rpt(RRc, RRv, min .1 max = Min(2 Val + .3, 10000);)
   WPPM_ft_conv = 1 – 0.75*WPPM_L/RRv + 0.0625*(WPPM_L/RRv)^3 ;
   WPPM_break_on_small = 1e-7;
   WPPM_L_max = 2*CeV(RRc, RRv);
   WPPM_th2_range = 55;
}

where RRc and RRv correspond to the crystallite radius and its numerical value, respectively. The total intensity is given by the weighted sum of all $I_{S}$ peaks, and is written as

(7) $$I\left(d^{*}\right)=\sum_{S}w_{s}\;I_{S}(d^{*})$$

where the summation covers all the possible sizes. This is performed by the macro histogram_fit.inc which is given in the Supplementary Note S3.

## 4. Results and Discussion

To verify the effectiveness of the size dependencies of the structural and microstructural parameters, experimental studies are essential to determine whether these second-order contributions truly matter in XRPD refinement. For this purpose, synchrotron data on a range of CeO_2_ samples with different size distributions, obtained from xerogels treated at different temperatures (300 °C, 500 °C, and 600 °C) [27], are analyzed. A key advantage of the Rietveld + TDS approach employed here is that peak-broadening effects can be treated independently from vibrational contributions, with the TDS scaling factor tied to the reduction in Bragg intensities through the Debye–Waller factor. This separation is difficult to achieve in real-space PDF analysis, where vibrational, static, and size effects are convoluted in peak widths, particularly for the first coordination shells.

**Figure 1:**
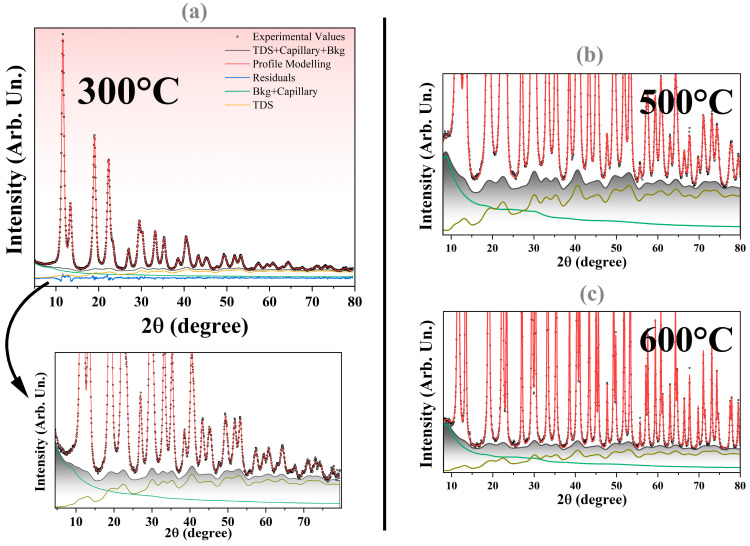
(**a**) Profile modeling of the XRD pattern for the sample measured at room temperature (300 °C). The black dots correspond to the experimental values. The profile modeling is given by the red line, the TDS + capillary + Chebyshev background is given by the gray line, residuals are displayed in blue, Chebyshev + capillary in green, and the TDS is given by the yellow line. The inset below shows an amplified version of the diffuse and background components. Profile modeling for the sample sintered at 500 °C (**b**) and (**c**) 600 °C.

Figure 1a shows the profile modeling for the sample treated at 300 °C. To accurately model the diffuse component, it is necessary to separate it from the background contributions arising from the capillary and air scattering, as illustrated in Figure 1a (lower panel). The TDS component is shown in yellow, and when combined with the capillary contribution, it forms the total background signal (indicated by the gray lines). The refined DW coefficient for Ce amounts to 0.62(2) Å^2^, which is significantly higher than the literature values of ~0.45 Å^2^ [45], while the DW coefficient for O refines to 0.89(7) Å^2^, again exceeding previously reported values [46]. This increase reflects larger static disorder contribution to the DW coefficient, becoming significant for the small crystallite sizes of CeO_2_ considered here [47,48]. Overall, the residuals exhibit only minor features, and the agreement factors remain low, with R_wp_ values of 2.59%, 2.31%, and 5.74% for the 300 °C, 500 °C, and 600 °C samples, respectively.

The oscillatory features of the TDS contribution are primarily located beneath the Bragg peaks, a behavior that is also observed for the other sintered powders shown in Figure 1b,c. These oscillations arise from correlated atomic displacements, from which the $\lambda_{r_{ss^{\prime }}}$ are extracted [40]. For each atomic pair type (Ce–Ce, Ce–O, and O–O), multiple correlation coefficients associated with different coordination shells must be considered. To validate the accuracy of the TDS modeling framework and to obtain reliable initial estimates of these coefficients, in silico MD simulations of bulk CeO_2_ were performed and discussed in the following section.

### 4.1. Local Dynamics Analysis via MD Simulations

For MD simulations, PBCs were employed to reproduce an effectively infinite crystal, thereby eliminating surface effects. This approach allows the intrinsic lattice dynamics and vibrational correlations of ceria to be isolated and directly compared with diffraction-derived quantities [20,48].

**Figure 2:**
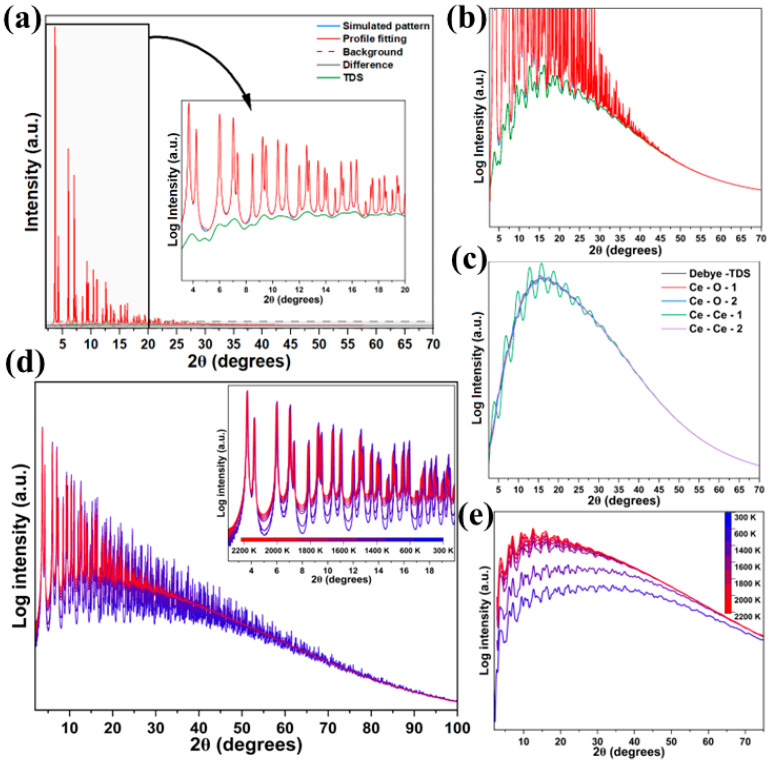
Simulated CeO_2_ XRD patterns and Bragg–TDS fits. (**a**) Total XRD pattern with refinement and TDS contribution (inset 4–20 degree region); (**b**) separation of Bragg and TDS (log scale); (**c**) Debye TDS and pairwise Ce–O and Ce–Ce scattering contributions; (**d**) temperature-dependent XRD patterns showing the evolution of Bragg component from 300 K to 2200 K; and (**e**) evolution of the TDS component with temperature (log scale).

To evaluate the influence of temperature on the diffraction response of ceria, MD simulations were carried out over a wide temperature range (300 to 2200 K). A representative refinement at 1400 K is shown in Figure 2a with an R_wp_ of 1.22% with residuals free of significant noise features. An enlarged view of the TDS contribution is shown in Figure 2b (green line). The TDS appears as a broad, low-intensity signal underneath the main Bragg peaks, extending into a long tail at high 2Ѳ values (high Q values). The decomposition of the TDS signal into coordination shell contributions (Figure 2c) shows that the Debye term governs this long-range tail, while the structured features beneath the Bragg peaks arise from correlated atomic displacements in the innermost coordination shells. Among the individual pair contributions, Ce-Ce correlations dominate the TDS intensity. This reflects the larger X-ray form factor of Ce relative to O, which increases the scattering weight of Ce-containing pairs in the diffuse component.

Figure 2d,e show the temperature dependence of the Bragg and TDS components. Thermal displacements attenuate the Bragg intensity via the Debye–Waller factor, $\exp\left(-B_{i}\;Q^{2}/(4\;\pi^{2})\right)$. The excellent agreement between MD and Rietveld + WPPM + TDS patterns across all temperatures confirms that the Sakuma TDS formalism accurately captures thermal disorder and yields physically meaningful displacement correlations (more details are included in Supplementary Note S4).

**Figure 3:**
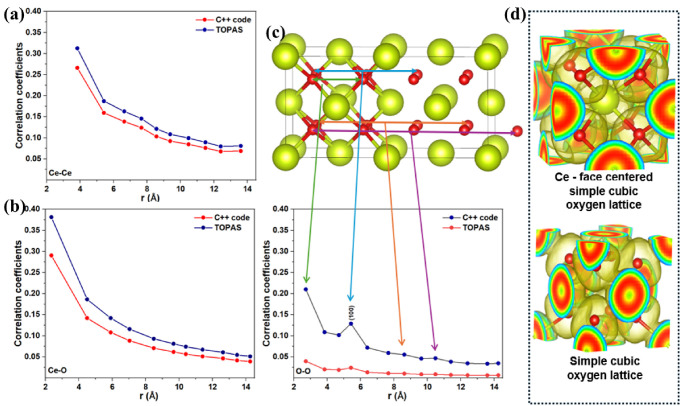
C++-derived and TOPAS-refined displacement correlation coefficients for (**a**) Ce–Ce, (**b**) Ce–O, and (**c**) O–O pairs in CeO_2_. The C++ code operates directly on the MD trajectories, extracting displacement correlations from the atomic positions as a function of time, whereas the TOPAS results are obtained by refining simulated XRD patterns generated from the same MD configurations. The center panel maps coordination shells from the fluorite lattice to the corresponding correlation features. (**d**) Visualization of the Ce face-centered cubic network surrounded by a simple cubic O lattice, and the isolated oxygen sublattice.

Displacement correlation coefficients $\lambda_{r_{ss^{\prime }}}$ were determined using two independent approaches: direct analysis of the MD trajectories employing a C++ code (Supplementary Note S1) and refinement of the same coefficients from diffraction data implemented in TOPAS. The resulting coefficients are shown in Figure 3a–c for Ce–Ce, Ce–O, and O–O pairs.

A comparison between correlation coefficients obtained from direct C++ trajectory analysis and those refined using TOPAS shows the largest discrepancies for O–O pairs, for which TOPAS yields systematically lower $\lambda_{r_{ss^{\prime }}}$ values and larger MSRDs. This behavior primarily reflects the much lower X-ray form factor of oxygen compared to cerium. In the low-Q limit, the X-ray scattering intensity scales approximately with the square of the atomic number (Z^2^). Since Z_O_ = 8 and Z_Ce_ = 58, the relative scattering strength per atom is approximately (8/58)^2^ ≈ 0.02. Thus, oxygen contributes only about 2% of the scattering intensity compared to cerium, leading to significantly reduced sensitivity to O–O displacement correlations in TDS refinement. The systematic underestimation of λ for O–O pairs is therefore attributed to this intrinsic sensitivity limitation rather than to deficiencies in the MD model. Nevertheless, despite minor non-monotonic temperature-dependent deviations, both approaches capture the same underlying physical trends, confirming the consistency between real-space MD analysis and reciprocal-space diffraction refinement.

A prominent feature of the O–O correlations is the pronounced peak at ~5.4 Å observed in both the C++ analysis and TOPAS refinement (Figure 3c), corresponding to second-neighbor O–O separations along ⟨100⟩. This non-monotonic behavior reflects the periodicity of the oxygen sublattice and is consistent with enhanced vibrational coherence along cube edges. In fluorite CeO_2_, the Ce atoms form a face-centered cubic framework, while the oxygen atoms occupy a simple cubic sublattice; along ⟨100⟩ directions, oxygen atoms are directly aligned without intervening Ce^4+^ ions. Consequently, O–O correlations along ⟨100⟩ exhibit a characteristic sequence, with a first-neighbor contribution at ~a/2, a strong second-neighbor peak at ~a, and weaker features at larger distances, indicating stronger coupling than along Ce-mediated directions. This anisotropy is supported by Figure 3d, where DFT-derived ELF isosurfaces show enhanced electronic connectivity along ⟨100⟩ within the oxygen sublattice and reduced overlap in directions involving Ce^4+^ ions, consistent with observations in other fcc-based systems [40].

**Figure 4:**
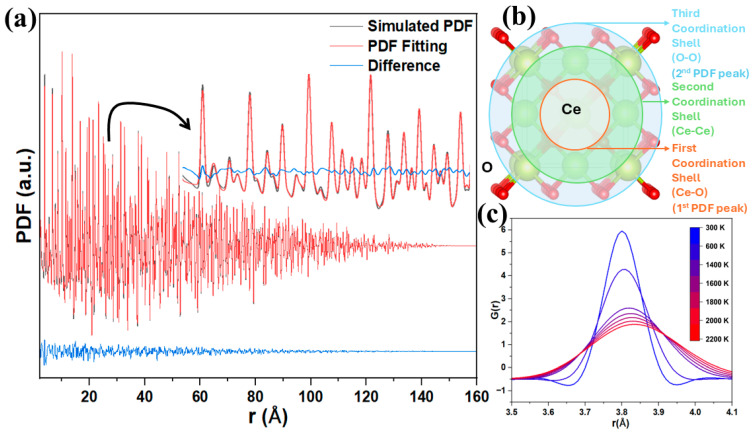
Simulated pair distribution function (PDF) and real-space structural interpretation for CeO_2_ nanocrystals. (**a**) Comparison between the simulated PDF, the TOPAS PDF refinement, and the corresponding difference curve. (**b**) Schematic representation of the Ce-centered coordination shells contributing to the first three PDF peaks. (**c**) Temperature-dependent Ce–Ce correlation functions illustrating thermal broadening and reduced structural coherence at elevated temperatures.

To further assess the displacement correlations obtained from MD simulations, real-space pair distributions were analyzed. The PDF is shown in Figure 4a, together with its small box fitted approach. The fitting resulted in an R_wp_ of 7.5% with some residual features observed due to the Fourier transformation (FT) truncation^2^. Figure 4b illustrates the structural origin of the first three PDF peaks by mapping the Ce-centered coordination shells in the fluorite lattice. The first-shell Ce–O, second-shell O-O, and third-shell Ce–Ce separations correspond directly to the dominant PDF features at low r, providing a structural reference for interpreting peak positions and changes in peak shape. As temperature increases, the PDF peaks exhibit a progressive broadening, which is directly reflected by the increase in the thermal variance $\sigma_{dyn}$ (assuming a Gaussian profile function), as illustrated for the first-neighbor Ce–Ce correlations in Figure 4c across the investigated temperature range. In addition to peak broadening, displacement correlations manifest quantitatively as peak symmetry and skewing, particularly at short interatomic distances [49]. Generally, the correlations are accounted for by [49,50]

(8) $${\sigma_{dyn}^{2}}^{\prime }=\sigma_{dyn}^{2}\left(1-\lambda_{r_{{ss}^{\prime }}}\right)$$

We observe that the truncation effects due to the limited $Q_{max}$ range employed in the FT has significantly affected the low-r region, introducing big deviations to the pattern. While this model captures qualitative trends, its quantitative reliability is limited by PDF truncation effects.

**Figure 5:**
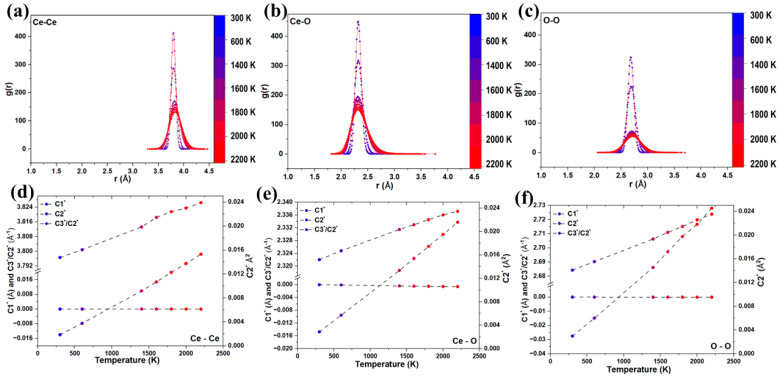
Temperature-dependent pair distribution behavior of CeO_2_. (**a**) Ce–Ce, (**b**) Ce–O, and (**c**) O–O radial distribution functions g(r) extracted from MD simulations from 300–2200 K, showing thermal broadening and peak attenuation with increasing temperature. (**d**–**f**) Corresponding first- and second-shell displacement parameters $C_{1}$, $C_{2}^{\prime }$, and correlation ratios $C_{3}^{\prime }/C_{2}^{\prime }$, highlighting the linear temperature dependence of local structural distortions for each atomic pair.

To overcome the limitations imposed by finite Q_max_ in PDF analysis, displacement correlations were evaluated directly in real space using radial distribution functions (RDFs) computed from the atomic trajectories. RDFs for all relevant atomic pairs are shown in Figure 5a–c. With increasing temperature, RDF peaks develop pronounced asymmetry, reflecting anharmonic atomic motion. The corresponding first-, second- and third-order cumulants are summarized in Figure 5d–f, with the third cumulant providing a direct measure of anharmonicity [51]. The temperature dependence of the second cumulant (MSRD) for each atomic pair was subsequently analyzed using an Einstein model, yielding effective vibrational frequencies, Einstein temperatures, force constants, and local thermal expansion coefficients. These Einstein model parameters for the Ce–O, Ce–Ce and O–O pairs are summarized in Table 1. Methods are described in Ref [40] and detailed profile expressions are given in Supplementary Note S5.

A clear physical hierarchy emerges from these results. The Ce–Ce pairs exhibit the highest effective force constants and Einstein temperatures, reflecting the rigidity of the cation sublattice, while the O–O pairs show lower values, indicating the greater flexibility and anharmonicity of the oxygen sublattice. The Ce–O pairs display intermediate behavior, consistent with their mixed ionic–covalent bonding character. These trends are consistent with the magnitude and temperature dependence of the displacement correlation coefficients obtained from both RDF and TDS analyses, confirming that the observed correlations reflect the underlying interatomic force landscape.

Overall, the combined TDS, PDF, and RDF analyses consistently show that the displacement correlation coefficients are governed by a limited set of structural and dynamical factors, including interatomic force constants, coordination, and pair separation. At short distances, the steepness of the interaction potential leads to strong restoring forces and, consequently, higher correlation coefficients, whereas correlations progressively weaken at larger separations. Coordination further modulates this behavior, as atoms in highly coordinated shells experience the collective influence of multiple neighbors. Within this framework, the correlation coefficients and the moments of the real-space pair distributions can be directly related to effective interatomic force constants through low-order expansions of the pair interaction potential. This establishes displacement correlations as physically grounded descriptors of local lattice dynamics, directly linking diffraction-derived observables and real-space displacement statistics. These concepts provide the basis for the following microstructural analysis of nanocrystalline ceria using experimental XRPD data.

### 4.2. Microstructural Analysis

**Figure 6:**
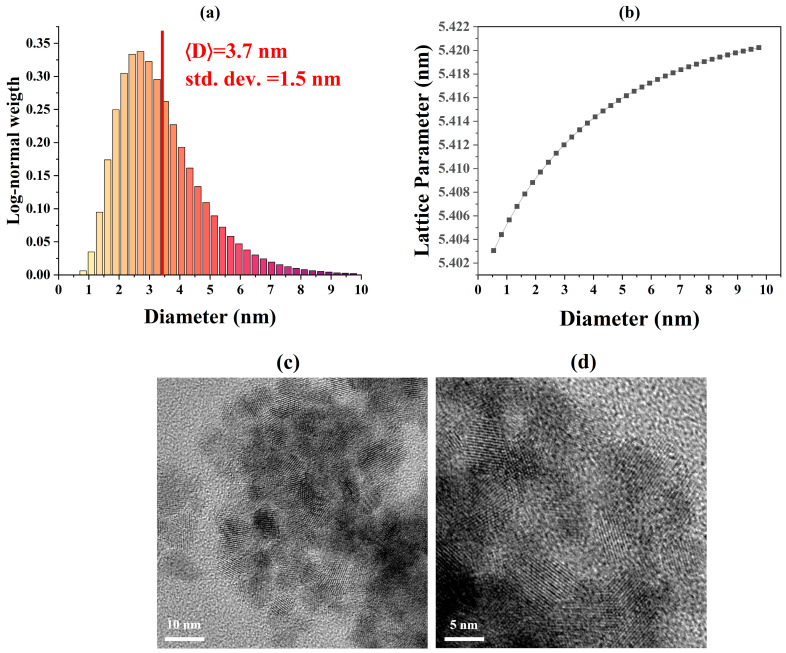
(**a**) Crystallite size distribution obtained via XRD profile modeling (the red line represents the average crystallite size). (**b**) Lattice parameter variation with crystallite diameter. High-resolution TEM micrographs of the 300 K CeO_2_ sample at (**c**) 400 K and (**d**) 500 K magnifications.

An additional challenge in the microstructural characterization of ceria nanoparticles concerns the dependence of the lattice parameter on the crystallite diameter D. To investigate this effect, new TOPAS macros were developed within the whole powder pattern modeling (WPPM) framework, as described in Section 3.1. Using this approach, the crystallite size distribution of the sample calcined at 300 °C was determined and is shown in Figure 6a. The analysis yields an average crystallite diameter of approximately 3.7 nm, with a relatively broad distribution of about 1.5 nm, modeled using a log-normal function. These results are in good agreement with the transmission electron microscopy (TEM) observations shown in Figure 6c,d [41].

The size dependence of the lattice parameter was modeled using a core–shell formalism inspired by electron energy-loss spectroscopy (EELS) observations [35], according to the following expressions:

(9) $$a\left(D\right)=a_{0}+\delta_{a}\;3.7\frac{f\left(h,D\right)}{4.4-0.7\;f(h,D)}$$

(10) $$f\left(h,D\right)=\frac{\left(\frac{D^{3}}{2^{3}}-{\left(\frac{D}{2}-h\right)}^{3}\right)}{\frac{D^{3}}{2^{3}}}$$

(11) $$h\left(D\right)=\frac{1}{2}D\;\left(1-{\left(1-f_{b}\right)}^{\frac{1}{3}}\;\right)$$

(12) $$f_{b}\left(D\right)=\frac{0.71\exp\left(-0.2\;D\right)}{1.31-0.91}$$

Here, f(h,D) represents the volume fraction of the shell relative to the core and depends on the shell thickness h, which in turn is governed by the Ce^4+^/Ce^3+^ ratio through the parameter $f_{b}$. The resulting lattice parameter evolution is shown in Figure 6b, where a clear lattice contraction is observed for crystallite diameters below approximately 10 nm. In this work, careful control of the synthesis conditions ensured that Ce^4+^ remained the predominant oxidation state even in the smallest nanospheres, with no contribution from surface adsorbates or hydroxyl species [27]. Under these conditions, defect-driven chemical expansion is suppressed, and the lattice response is consistent with surface-stress-driven contraction at small sizes, resembling behavior commonly observed in metallic nanoparticles [52]. This provides a consistent explanation for the experimentally observed size-dependent lattice behavior.

Most of the literature reports interpret lattice parameter variations in ceria nanoparticles as the result of two competing contributions: defect-driven chemical expansion and surface chemistry. Lattice expansion is commonly attributed to an increased fraction of Ce^3+^ relative to Ce^4+^, owing to the larger ionic radius of Ce^3+^, particularly at nanoparticle surfaces where reduced coordination and oxygen vacancy formation are more likely [35]. As a consequence, many studies report lattice expansion with decreasing crystallite size [15,41,43,53,54,55,56]. In addition, adsorbates and hydroxyl species present at the surface are also associated with lattice expansion as particle size decreases, and can dominate the size-dependent lattice behavior at small crystallite sizes [19]. However, the lattice parameter of ceria nanoparticles reflects a balance between defect-induced expansion and compression arising from surface stress, which can lead to lattice contraction at small particle sizes when defect and surface chemistry contributions are weak or absent [15].

Importantly, the analysis performed here differs from the majority of prior XRPD studies, which infer size-dependent lattice trends by comparing separate synthesis batches with different average crystallite sizes. In such comparisons, variations in the defect chemistry and hydration state are often correlated with the particle size, making it difficult to decouple intrinsic size effects from chemical expansion. In contrast, the histogram-based WPPM approach employed here extracts the lattice parameter–size coupling directly within a single specimen, thereby reducing batch-to-batch variability and enabling isolation of the intrinsic elastic contribution to the size-dependent lattice response. As a result, the lattice response is dominated by surface stress acting on an intrinsically stiff but size-constrained lattice. However, under realistic catalytic or environmental conditions, surface chemistry, particularly oxygen vacancy formation and adsorbate interactions, can substantially change this behavior. To explicitly assess how surface reduction and hydration alter the response of ceria nanoparticles, reactive MD simulations were carried out to gain insights into the underlying mechanisms.

#### ReaxFF Simulations of CeO_2_

**Figure 7:**
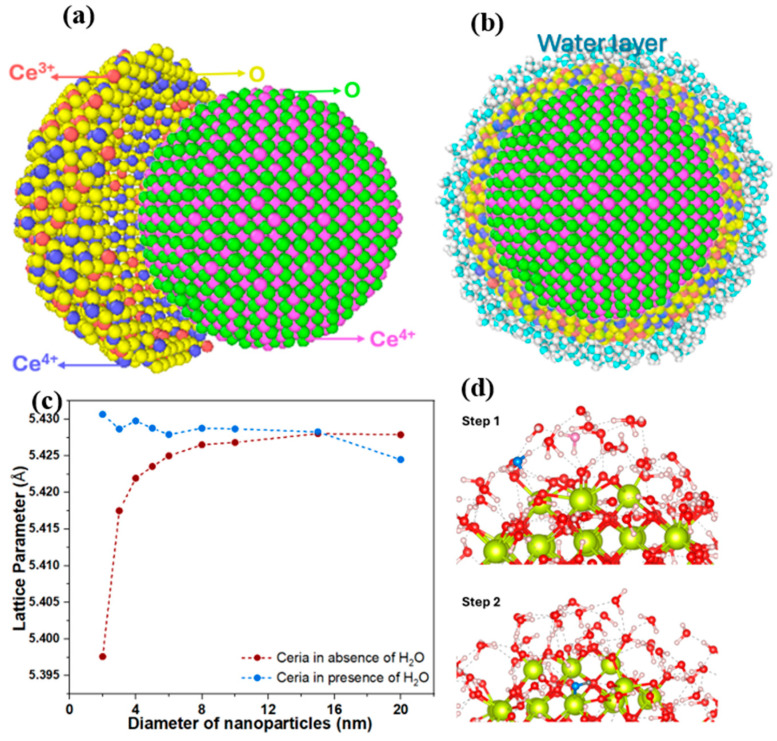
(**a**) Core–shell model of CeO_2_, a core with stoichiometric CeO_2_ and a shell containing 10% vacancies of the surface oxygens. (**b**) A hydrated CeO_2_ nanoparticle. (**c**) The plot represents the variation in the lattice parameter in case of CeO_2_ with vacancies in absence of water and in presence of water and (**d**) represents the mechanism in which water is adsorbed onto the surface of ceria.

In this case, reactive MD simulations were employed to directly investigate how surface oxygen vacancies control the structural response of CeO_2_ nanoparticles. The simulated core–shell nanoparticle model consists of a stoichiometric CeO_2_ core, and a vacancy-rich surface shell as illustrated in Figure 7a. In addition, to emulate water adsorption expected under realistic catalytic conditions, a thin layer of water was introduced around the nanoparticle, as shown in Figure 7b. All details are given in Section 2.2.2.

Our simulations show that CeO_2_ nanoparticle vacancy-rich surface shells exhibit lattice contraction at small sizes under vacuum conditions (see Figure 7c, red curve). Although oxygen vacancies locally relax the surrounding lattice and promote chemical expansion in their immediate vicinity, the net lattice response in this regime is contractive, indicating that compressive surface effects outweigh vacancy-driven expansion. This behavior is consistent with the bulk lattice-dynamical analysis presented in Section 4.1, which indicates a relatively stiff Ce–O framework, implying that surface-induced elastic effects can dominate over localized defect relaxation at small particle sizes. This contraction is most pronounced for the smallest particles, where the surface-to-volume ratio is highest and surface contributions have the strongest influence on the average lattice parameter. As particle size increases, the relative contribution of the surface shell decreases, and the lattice parameter progressively approaches the bulk value (5.427 Å). These results demonstrate that surface oxygen vacancies do not necessarily result in lattice expansion under dry conditions.

Such chemically driven relaxation processes involve bond breaking and formation and cannot be captured by non-reactive force fields or harmonic lattice-dynamical models. To assess the influence of surface chemistry under hydrated conditions, water–ceria interactions were investigated using reactive MD simulations. As shown in Figure 7c (blue curve), hydration reverses the size dependence of the lattice parameter by suppressing surface-stress-dominated contraction and inducing lattice expansion at small diameters. Water preferentially adsorbs at oxygen vacancies and under-coordinated Ce sites (step 1), where dissociative adsorption leads to hydroxyl formation and partial reoxidation of Ce^3+^ to Ce^4+^ (step 2), as illustrated schematically in Figure 7d (a quantitative analysis on the mechanism is provided in Supplementary Note S6). The relaxation of surface stress and stabilization of expanded lattice configurations are due to chemical restructuring of the hydroxylated surface, with the effect being strongest for the smallest nanoparticles. This trend shows the increasing dominance of surface contributions as the surface-to-volume ratio increases, which amplifies the effect of adsorbate-induced relaxation on the average lattice parameter. These results indicate that the observed lattice parameter trends in ceria nanoparticles are governed by the interplay between surface reduction, surface stress, and adsorbate-induced relaxation, in agreement with experimental observations and prior literature [15]. Thus, lattice parameter variations in nanoceria emerge as a chemically driven surface phenomenon that becomes increasingly dominant at small particle sizes. These lattice parameter variations can be used as a gauge to detect the saturation of water molecules on the nanoparticle surface during in operando experiments.

## 5. Conclusions

This work presents a diffraction–simulation framework for probing crystallite size effects, lattice dynamics, and surface chemistry in nanocrystalline CeO_2_. By combining synchrotron XRPD with WPPM and TDS analysis, we demonstrate that diffuse scattering provides quantitative information on correlated atomic motion, which can be reliably extracted using the Sakuma formalism and validated against MD simulations. Since TDS becomes significant for small nanocrystals, as observed in this case, it is essential to obtain an accurate line profile fitting where microstructural strain and size effects are modeled. The study further reveals that the lattice parameter trends in ceria nanoparticles are governed by both size and surface environment. Vacancy-rich surfaces induce lattice contraction at small sizes, while water adsorption leads to surface hydroxylation and lattice expansion. These results show that lattice expansion in nanoceria cannot be uniquely attributed to oxygen vacancies but is strongly modulated by surface chemistry. Unlike prior studies that treat diffraction, lattice dynamics, or surface effects separately, this work presents a unified, experimentally grounded framework linking size distributions, atomic correlations, and surface processes, with direct relevance to oxide nanocatalysts. The framework established here provides a foundation for future studies on doped ceria systems and for coupling in situ diffraction with reactive simulations under catalytic conditions, enabling a more comprehensive understanding of structure–chemistry–function relationships in oxide nanomaterials.

## Figures and Tables

**Table 1:** Einstein model parameters for the Ce–Ce, Ce–O and O–O pairs in CeO_2_ extracted from the cumulant analysis of RDFs.

Pair	ωₑ (10^13^ s^−1^)	θₑ (K)	a (eV Å^−2^)	α (10^−6^ K^−1^)
Ce–Ce	8.84 ± 0.04	675 ± 3	11.62 ± 0.1	5.83 ± 0.08
Ce–O	7.54 ± 0.02	576 ± 2	8.46 ± 0.05	3.48 ± 0.2
O–O	7.04 ± 0.05	538 ± 2	7.38 ± 0.09	7.79 ± 0.2

## Data Availability

The original contributions presented in this study are included in the article/Supplementary Material. Further inquiries can be directed to the corresponding authors.

## Supplementary Materials

The following supporting information can be downloaded at: https://www.mdpi.com/article/10.3390/nano16050333/s1. References [57,58,59,60,61] are cited in the supplementary materials.
