# Supplementary material for: XRD and Molecular Dynamics Insights into Lattice Behavior of Oxide Nanocatalysts: The Case of CeO2
Source: Nanomaterials (Basel). 2026 Mar 6;16(5):333. doi: 10.3390/nano16050333 (PMC12986646; doi:10.3390/nano16050333)
Supplement: Supplementary file 1 [file nanomaterials-16-00333-s001.zip › nanomaterials-4185273-Supplementary Notes.pdf]

XRD and Molecular Dynamics Insights into Lattice Behavior of Oxide Nanocatalysts: The Case of CeO_2

Sirisha Subbareddy [1], Marcelo Augusto Malagutti [1,*], Himanshu Nautiyal [2], Narges Ataollahi [1] and Paolo Scardi [1,*]

[1] Department of Civil, Environmental, and Mechanical Engineering, University of Trento, 38123 Trento, Italy; sirisha.subbareddy@unitn.it (S.S.); narges.ataollahi@unitn.it (N.A.)

[2] Department of Physics, School of Physical and Biological Sciences, Manipal University Jaipur, Jaipur 303007, Rajasthan, India; himanshu.nautiyal@jaipur.manipal.edu

* Correspondence: marcelo.malagutti@unitn.it (M.A.M.); paolo.scardi@unitn.it (P.S.)

Academic Editor: Gregory M. Odegard

Received: 16 February 2026
Revised: 2 March 2026
Accepted: 4 March 2026
Published: 6 March 2026

Copyright: © 2026 by the authors. Submitted for possible open access publication under the terms and conditions of the Creative Commons Attribution (CC BY) license.

Supplementary Note S1

To provide physically grounded starting values for the displacement-correlation coefficients used in the Sakuma TDS model, a dedicated C++ code was developed to extract correlation coefficients directly from MD trajectories. The code reads a trajectory consisting of successive atomistic configurations ("frames"), computes time-averaged atomic positions, evaluates atomic displacements relative to these averages, and then quantifies correlated motion for selected atomic pair types (Ce–Ce, Ce–O, and O–O). Pair correlations are finally grouped according to coordination shells by assigning each pair to a distance bin defined from a reference configuration (typically the first frame), thereby producing shell-resolved correlation coefficients $\lambda_{r_{ss'}}$, suitable for use as initial parameters in TOPAS TDS refinement.

For each atom i, the time-averaged position is computed from the trajectory as

$$\langle \boldsymbol{r}_i \rangle = \frac{1}{T}\sum_{t=1}^{T} r_i(t) \tag{1}$$

where T is the number of frames and $\mathrm{r}_i(t)$ is the Cartesian position of atom i at time t. Atomic displacements are then defined relative to the time-averaged position as

$$\Delta\langle \boldsymbol{r}_i \rangle = r_i(t) \; - \; \langle \boldsymbol{r}_i \rangle \tag{2}$$

From these displacements, the mean-square displacement (MSD) for species s is evaluated by averaging over atoms of type $s$and over time:

$$\mathrm{MSD_s} = \langle |\Delta \boldsymbol{r}_i(t)|^2 \rangle_{i\in s,t} \tag{3}$$

For each atomic pair type (s, s'), the displacement cross-correlation (dot product) is computed frame-by-frame as

$$C_{ij}(t) = \Delta \boldsymbol{r}_i(t) \cdot \Delta \boldsymbol{r}_j(t) \tag{4}$$

and then averaged over all pairs (i, j) that belong to the same pair type and coordination-shell bin (see below), as well as over time:

$$\langle \Delta \boldsymbol{r}_s \cdot \Delta \boldsymbol{r}_{s'} \rangle_r = \left\langle C_{ij}(t) \right\rangle_{(i\in s,\, j\in s',\, r_{ij}\in r),\, t} \tag{5}$$

Using these quantities, the shell-resolved displacement correlation coefficient is defined consistently with Eq. (1) in the main text as

$$\lambda_{r_{ss'}} = \frac{\langle \Delta r_s \cdot \Delta r_{s'} \rangle}{\langle |\Delta r_s|^2 \rangle + \left\langle |\Delta r_{s'}|^2 \right\rangle} \tag{6}$$

To obtain a coordination-shell decomposition, each pair is assigned to a shell/bin based on its distance in a reference configuration. The reference pair distance is computed as

$$r_{ij}^{\mathrm{ref}} = |r_i^{\mathrm{ref}} - r_j^{\mathrm{ref}}| \tag{7}$$

and the corresponding shell index is determined by a distance binning rule of the form

$$\mathrm{bin}(r) = \left\lfloor \frac{r_{ij}^{\mathrm{ref}}}{\Delta r} \right\rfloor \tag{8}$$

where Δr is the bin width (set in the code). All pair-correlation accumulators are stored as a function of (i) pair type (s, s') and (ii) bin index, yielding shell-resolved averages of r, $\langle \Delta \mathbf{r}_s \cdot \Delta \mathbf{r}_{s'} \rangle_r$, and finally $\lambda_{r_{ss'}}$. The resulting outputs provide (i) time-averaged coordinates, (ii) shell-resolved correlation coefficients for each pair type, and (iii) supporting pair-distance statistics used to map the extracted $\lambda_{r_{ss'}}$values onto coordination shells in the fluorite structure.

Note: The code used here will be made available from the authors upon request.

Supplementary Note S2

The code was developed to generate the necessary files and macros for using the Sakuma expression of the thermal diffuse scattering (TDS) in TOPAS. It currently works for cubic, tetragonal, and orthorhombic structures (i.e., $\alpha = \beta = \gamma = 90°$). The theory is derived in https://doi.org/10.1021/acs.cgd.3c01507 and https://doi.org/10.1107/S1600576724010756.

In summary, the code opens a .cif file to read the structure, it expands the unit cell in all the a,b,c axis direction of the structure, calculate the number of pair distances in each shell (Z values), and also the pair distances for each shell in terms of the lattice parameters.

It is recommended that an initial refinement be performed without including TDS contributions, ensuring that the TOPAS .inp file is properly set up. The TDS contribution can then be introduced by copying the relevant macro snippets generated by the code into the refined .inp file.

The code and a step-by-step tutorial illustrating its use (demonstrated for a simulated Pd sphere in vacuum) are not publicly released but will be made available from the authors upon request.

Supplementary Note S3

The macro "LPSizeVariationSTR" in the file *histogram_fit.inc* implements a size-resolved whole powder pattern modelling (WPPM) approach for CeO_2 nanoparticles, in which each diffraction pattern is expressed as the sum of contributions from crystallites of different diameters. Each crystallite size class is treated explicitly, with structural, microstructural, and intensity normalization terms calculated as functions of the crystallite diameter D.

Crystallites are assumed to be spherical, and the crystallite radius is defined as

$$R = D/2 \tag{9}$$

The contribution of each crystallite size bin is weighted using a log-normal size distribution,

$$w(D) = \frac{1}{\sqrt{2\pi}\, D\, \sigma} \exp\left[-\frac{(\ln D - \mu)^2}{2\sigma^2} \right] \tag{10}$$

where μ and σ are the mean and standard deviation of the logarithmic diameter. This weighting is applied directly to the scale factor to ensure correct normalization of the total diffracted intensity.

To account for surface-related effects, the macro estimates the fraction of surface unit cells using a geometrical shell model. An empirical size-dependent bulk fraction is defined as

$$f_b(D) = \frac{0.71\ \exp(-0.2D)}{1.31-0.91} \tag{11}$$

from which an effective shell thickness is calculated as

$$h = \frac{D}{2}\left[1-(1-f_b)^{1/3}\right] \tag{12}$$

The corresponding surface volume fraction is then given by

$$f = \frac{R^3-(R-h)^3}{R^3} \tag{13}$$

The lattice parameter of a crystallite with diameter $D$is expressed as an explicit function of the surface fraction,

$$a(D) = a_0 + \Delta a_{0,max}\ \frac{3.7\,f}{4.4-0.7\,f} \tag{14}$$

re $a_0$is the bulk lattice parameter and $\Delta a_{0,\mathrm{max}}$ represents the maximum lattice parameter deviation induced by size effects. This formulation ensures a smooth transition between bulk-like and surface-dominated regimes.

For each crystallite size, the number of unit cells contained within a spherical particle is calculated as

$$N_{\mathrm{uc}}(D) = \frac{\pi}{6}\ \frac{D^3}{[a(D)/10]^3} \tag{15}$$

where the factor of 10 converts the lattice parameter from ångströms to nanometres. Assuming the fluorite structure stoichiometry, the number of atoms per crystallite is given by

$$N_{\mathrm{Ce}} = 4N_{\mathrm{uc}}, \qquad N_{\mathrm{O}} = 8N_{\mathrm{uc}}$$

The crystal structure of CeO_2 is defined in space group *Fm*-3*m*, with Ce atoms occupying the 4a sites $(0, 0, 0)$and O atoms occupying the 8c sites $\left(\frac{1}{4},\frac{1}{4},\frac{1}{4}\right)$. Atomic displacement parameters for Ce and O are refined independently and assumed to be independent of crystallite size within each size bin. Structure factors are calculated point-by-point to ensure consistency with the WPPM formalism.

Crystallite size broadening is modelled using the spherical WPPM domain model. Microstrain broadening is included through Gaussian and Lorentzian components. The root-mean-square Gaussian microstrain is defined as

$$\varepsilon_{\mathrm{rms}}^{\mathrm{G}} = \sqrt{\left|e_{0,\mathrm{G}}^2\ \frac{1.6\,f(1-f)}{2.6-0.4f}\right|} \tag{16}$$

while the Lorentzian microstrain component is defined as

$$\varepsilon_{\mathrm{rms}}^{\mathrm{L}} = \left|e_{0,\mathrm{L}}\right| \tag{17}$$

Both microstrain components are converted to full widths at half maximum using standard WPPM relations and passed to the TOPAS strain-broadening formalism.

Finally, the diffraction intensity contribution of each crystallite size bin is scaled as

$$I(D) \propto \text{scale} \cdot w(D) \frac{N_{\text{uc}}(D)\, \lambda^2}{16\pi\, a(D)^3} \tag{18}$$

ensuring correct absolute normalization and a quantitative representation of the full crystallite size distribution in the calculated diffraction pattern.

Supplementary Note S4

XRD Patterns at 300 K, 600 K, 1600 K, 1800 K, 2000 K and 2200 K.

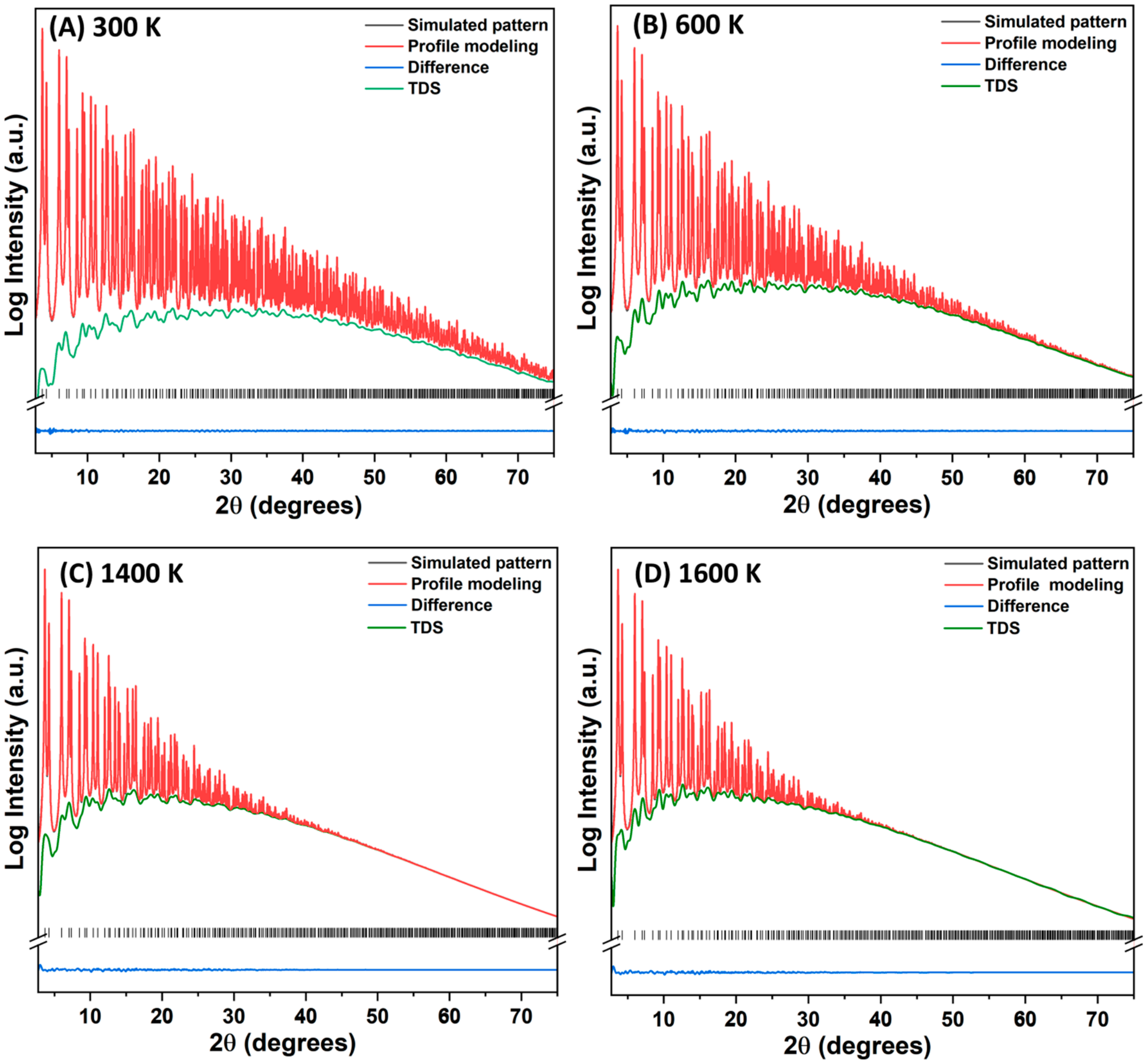

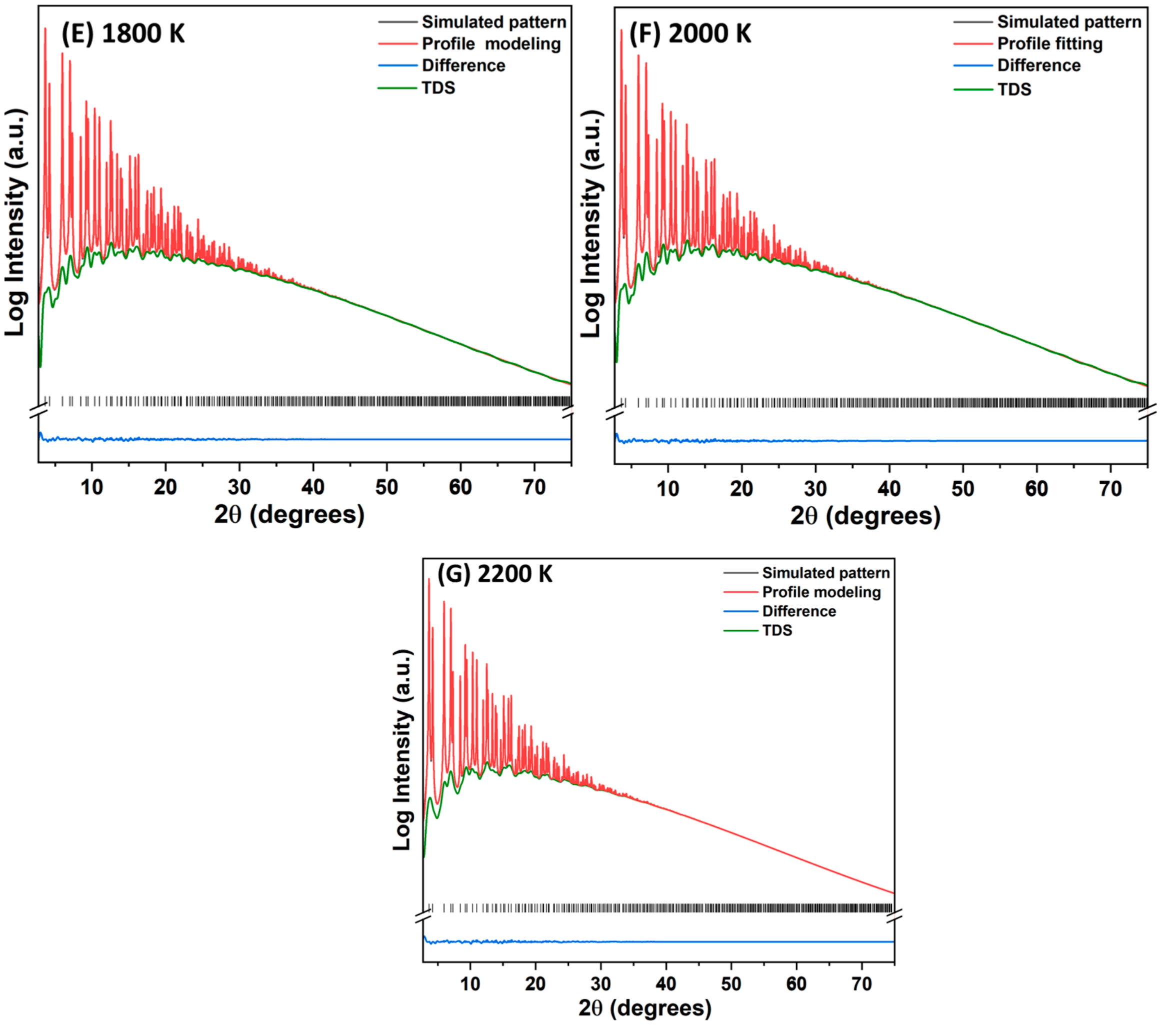

Figure S1: Total scattering patterns showing the contribution of the Bragg and the TDS components at (A) 300 K (R_{wp} = 0.037%), (B) 600 K (R_{wp} = 0.82%), (C) 1400 K (R_{wp} = 1.22%), (D) 1600 K (R_{wp} = 1.22%), (E) 1800 K (R_{wp} = 2.62%), (F) 2000 K (R_{wp} = 2.71%) and (G) 2200 K (R_{wp} = 1.27%)

Figure S1 presents the simulated XRD patterns of CeO_2 at 300, 600, 1400, 1600, 1800, 2000, and 2200 K, highlighting the evolution of both Bragg and thermal diffuse scattering (TDS) components. Across all temperatures, the Bragg peak positions remain effectively unchanged, consistent with the fixed-cell PBC model employed, while their intensities progressively decrease due to Debye–Waller attenuation. In contrast, the TDS background systematically increases with temperature and becomes more prominent at high 2θ, reflecting the enhanced vibrational disorder and larger atomic mean-square displacements. The separation between Bragg and diffuse contributions is shown for each temperature to illustrate the redistribution of scattering intensity. Despite these temperature-induced changes, all refinements remain stable, and the residuals exhibit no significant systematic deviations, confirming the robustness of the TDS modelling across the full thermal range.

Supplementary Note S5

The displacement correlation coefficients $\lambda_{r_{ss'}}$, derived from the MD trajectories are directly related to the mean-square displacements (MSDs) and mean-square relative displacements (MSRDs), which control the width and shape of real-space pair correlations. To examine how these correlations manifest in real space, and to assess their temperature dependence and physical origin,

complementary analyses based on the pair distribution function (PDF) and radial distribution functions (RDFs) were performed.

Following established PDF analysis procedures, the PDF peaks were modelled in TOPAS using Gaussian-based profile functions. While this functional form does not represent a fundamental physical law, it provides a widely adopted and practical approximation for describing local pair distributions in crystalline and nanocrystalline materials. Within this framework, the total variance of a PDF peak, σ^2, was decomposed into a thermal (dynamic) contribution and a static strain contribution according to

$$\sigma^2 = \sigma_{dyn}^2 + \delta_G^2\, r^2 \tag{19}$$

where σ_{dyn}^2represents the r-independent thermal component and $\delta_G$corresponds to the root-mean-square strain (RMSS) associated with a Gaussian strain distribution.

To account for peak asymmetry and deviations from an ideal Gaussian shape at larger interatomic distances, the PDF peaks were modelled using the following generalized expression:

$$g(r) = \frac{A}{\sigma_1\sqrt{2\pi}} \exp\left[-\frac{1}{2}\left(\frac{r-R}{\sigma_1}\right)^2 \left(1 - \frac{(r-R)}{R}\frac{\sigma_2^2}{\sigma_1^2}\right)\right] \tag{20}$$

where A is the peak amplitude, R the average interatomic distance, and $\sigma_1$and $\sigma_2$are refinable width parameters. Setting $\sigma_2 = 0$ recovers the Gaussian limit. The displacement correlation coefficients $\lambda_{r_{ss'}}$were subsequently extracted from the second moment of the fitted $g(r)$distributions.

Supplementary Note S6

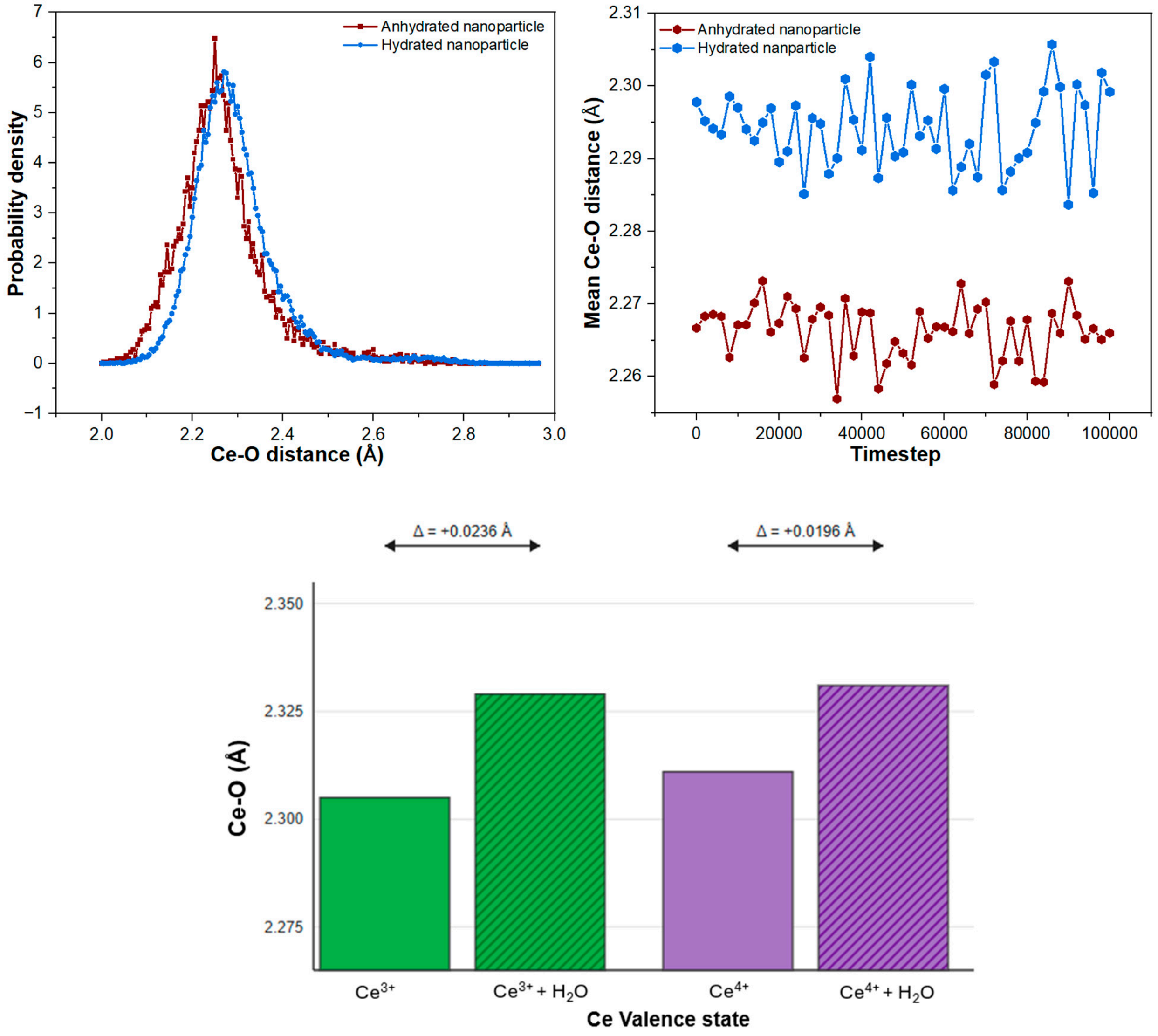

Figure S2: (a) Nearest-neighbour Ce-O bond-length distributions for anhydrous (brown) and hydrated (blue) nanoparticles. (b) Time evolution of the mean nearest-neighbour Ce-O bond length for anhydrous (brown) and hydrated (blue) nanoparticles. (c) Mean Ce–O bond lengths for Ce^{3+} and Ce^{4+} sites under dry and hydrated conditions, showing systematic bond elongation of $\Delta\langle r\rangle$ = +0.0236 Å for Ce^{3+}–O and +0.0196 Å for Ce^{4+}–O upon water adsorption. Error bars represent one standard deviation.

To quantitatively establish the microscopic mechanism underlying the lattice expansion observed upon hydration, nearest-neighbour Ce-O bond-length distributions were extracted from the ReaxFF molecular dynamics trajectories for both anhydrous and hydrated 2 nm CeO_2 nanoparticles. For each timestep, the nearest oxygen neighbour within a 3 Å cutoff was identified for every Ce atom, and the bond-length statistics were accumulated over the equilibrated portion of the trajectory.

The resulting probability density distributions are shown in Fig. S2a. Hydration induces a systematic rightward shift of the Ce-O bond-length distribution relative to the anhydrous nanoparticle. The main

peak position increases by approximately 0.02-0.03 Å for the 2 nm particle, indicating bond elongation associated with hydroxylation and partial surface reoxidation.

The temporal evolution of the mean nearest-neighbour Ce-O distance is shown in Fig. S2b. After equilibration, both systems exhibit stable fluctuations around well-defined mean values. The hydrated nanoparticle consistently maintains a larger average Ce-O bond length than the anhydrous system across the entire trajectory. The time-averaged bond elongation $\Delta\langle r_{Ce\text{-}O}\rangle \approx 0.025–0.030$ Å provides a quantitative microscopic metric linking surface chemical restructuring to the macroscopic lattice expansion observed in the size-dependent analysis.

These results confirm that hydroxylation modifies the local coordination environment of surface cerium atoms, leading to bond elongation and, consequently, an increase in the effective lattice parameter. The bond-level shift is consistent in magnitude with the lattice expansion extracted from structural refinement, supporting the interpretation that hydration-driven chemical relaxation governs the observed structural response. To further quantify the valence-state dependence of this bond elongation, Ce-O bonds were separated by Ce oxidation state (Ce^{3+} and Ce^{4+}) and compared between dry and hydrated conditions, as shown in Fig. S2c. Ce^{3+}-O bonds elongate by $\Delta\langle r\rangle = +0.0236$ Å and Ce^{4+}-O bonds by +0.0196 Å upon hydration, confirming that both Ce oxidation states contribute to the macroscopic lattice expansion, with Ce^{3+} sites showing the larger structural response consistent with their greater ionic radius and weaker Ce-O bond covalency.

The microscopic influence of Ce^{3+}/Ce^{4+} valence state changes on the electronic connectivity of the Ce–O sublattice can be understood in the context of established DFT results from the literature. Ce^{4+} ($4f^0$) forms shorter, more covalent Ce–O bonds in bulk CeO_2, while Ce^{3+} ($4f^1$) — with its larger ionic radius (1.143 Å vs. 0.97 Å for Ce^{4+} in eight-fold coordination[1]) — forms longer and more ionic Ce–O bonds at reduced ceria surfaces, as demonstrated by DFT+U calculations[2],[3]. The localisation of the excess 4f electron on Ce^{3+} sites reduces the charge transfer to neighbouring oxygen atoms, weakening the Ce–O bond and reducing the effective force constant; this 4f localisation and its structural consequences have been systematically characterised as a function of the Hubbard U parameter using DFT+U[4],[5]. This bond weakening is reflected in our ReaxFF trajectories as a systematic elongation of the Ce–O nearest-neighbour distance upon hydration ($\Delta\langle r_{Ce\text{–}O}\rangle \approx +0.024$–0.028 Å, Figure S2), quantitatively consistent with the DFT-predicted structural response to Ce^{3+} formation. The DFT literature therefore provides direct electronic-structure grounding for the bond elongation and lattice expansion trends observed here, linking the macroscopic structural response to the microscopic change in Ce 4f occupancy and Ce–O bond covalency upon partial reoxidation.

References

1. Shannon, R.D. Revised Effective Ionic Radii and Systematic Studies of Interatomic Distances in Halides and Chalcogenides. *Acta Crystallogr. Sect. A* **1976**, *32*, 751–767, doi:10.1107/S0567739476001551.

2. Nolan, M.; Grigoleit, S.; Sayle, D.C.; Parker, S.C.; Watson, G.W. Density Functional Theory Studies of the Structure and Electronic Structure of Pure and Defective Low Index Surfaces of Ceria. *Surf. Sci.* **2005**, *576*, 217–229, doi:10.1016/j.susc.2004.12.016.

3. Nolan, M.; Parker, S.C.; Watson, G.W. The Electronic Structure of Oxygen Vacancy Defects at the Low Index Surfaces of Ceria. *Surf. Sci.* **2005**, *595*, 223–232, doi:10.1016/j.susc.2005.08.015.

4. Loschen, C.; Carrasco, J.; Neyman, K.M.; Illas, F. First-Principles LDA+U and GGA+U Study of Cerium Oxides: Dependence on the Effective U Parameter. *Phys. Rev. B* **2007**, *75*, 035115, doi:10.1103/PhysRevB.75.035115.
5. Castleton, C.W.M.; Kullgren, J.; Hermansson, K. Tuning LDA+U for Electron Localization and Structure at Oxygen Vacancies in Ceria. *J. Chem. Phys.* **2007**, *127*, doi:10.1063/1.2800015.
